# D-Tagatose as a Sucrose Substitute and Its Effect on the Physico-Chemical Properties and Acceptability of Strawberry-Flavored Yogurt

**DOI:** 10.3390/foods8070256

**Published:** 2019-07-12

**Authors:** Damir D. Torrico, Jennifer Tam, Sigfredo Fuentes, Claudia Gonzalez Viejo, Frank R. Dunshea

**Affiliations:** 1 Faculty of Veterinary and Agricultural Sciences, School of Agriculture and Food, The University of Melbourne, Parkville, VIC 3010, Australia; 2 Department of Wine, Food and Molecular Biosciences, Faculty of Agriculture and Life Sciences, Lincoln University, Lincoln 7647, New Zealand

**Keywords:** tagatose, sugar reduction, yogurt, physico-chemical, acceptability

## Abstract

Sugar not only provides the desirable sweetness but its reduction can also alter the physico-chemical properties of foods. The objective of this study was to evaluate the effects of tagatose as a sugar substitute on selected physico-chemical properties and sensory acceptability of strawberry-flavored yogurts. Six yogurt samples with decreasing concentrations of sucrose (8.50 to 1.70 g/100 g) and increasing concentrations of tagatose (0.00 to 9.24 g/100 g) were evaluated. Physico-chemical tests (pH, lactic acid (%), °Brix, water-holding capacity (WHC), viscosity, and color) were conducted to examine the quality and shelf-life of yogurts during 28 days of storage at 4 °C. An acceptability test (*n* = 55) was conducted to evaluate the sensory characteristics of yogurts. Sucrose reductions by the replacement of up to 80% tagatose showed marginal effects on the selected physico-chemical properties; however, the loss of red color (a*) and increase in yellowness (b*) of the tagatose-substituted samples were significant. Strawberry yogurts with tagatose replacements had similar acceptability scores for all attributes. Sucrose reduction showed a positive effect on the purchase intent of the strawberry yogurts (an increase of 3–30%). These findings can be used to understand the effects of tagatose/sucrose formulations on the acceptability and physico-chemical properties of yogurts.

## 1. Introduction

Being overweight or obese due to the excessive consumption of unhealthy foods and the increasing adoption of a sedentary lifestyle are becoming a growing human health problem worldwide [1]. Progressively, consumers are more concerned about the health-related implications of consuming high-calorie foods such as the occurrence of type 2 diabetes and cardiovascular diseases [2]. Therefore, consumers are looking for changes in their diets and lifestyles to avoid becoming overweight, which leads to the growing demand for healthy food products [3]. Within this context, the reduction of sugar consumption in the world population is a critical factor toward combating obesity [4]. However, the food industry faces a challenging task because sugar not only provides the desirable sweet taste, but its reduction can also alter the physico-chemical properties of foods [5], and potentially its taste and preference. 

Yogurt, which exhibits healthful and desirable sensory attributes, is increasingly being consumed worldwide [6,7]. Over the past few years, a great variety of yogurt flavors have been developed to supply the consumers’ demand for yogurt products. Therefore, the yogurt industry is striving to develop yogurt products that deliver additional health benefits. The development of symbiotic yogurt is emerging due to the synergistic health effects of probiotics and prebiotics [8]. At the same time, the development of low-sugar and low-calorie yogurt products has been the focus of the food industry since the discovery of alternative sweeteners [9]. Few studies have explored the effects of sugar reductions on the sensory perception of yogurt [10], albeit with the dose–response relationship of sucrose on other food matrices having been studied broadly [11]. Sucrose possesses bulking properties, which not only contribute to the sweetness but also to the total solids, texture, body, viscosity, and moisture retention in yogurts [9,12]. Past studies have shown that sugar reductions in foods can produce a reduction in product acceptability [13]. Sugar replacers or substitutes, also known as alternative sweeteners, are food additives that mimic the sweetness of sugar without having higher amounts of calories [1]. The food industry is increasingly replacing sugar with alternative sweeteners in traditional food products [14]. Sugar reduction through the substitution of sucrose by sugar replacers is the most common strategy to produce sugar-reduced products. 

Recently, an emerging sweetener known as D-tagatose has drawn much attention in the food industry. D-Tagatose, commonly referred to as tagatose, is a stereoisomer of fructose that inverts its hydroxyl and hydrogen group on the fourth carbon [15]. It is a rare sugar that provides a sweet sucrose-like taste, but with less intensity (90–92% of sucrose sweetness) and less than a half of the calories (1.5 kcal/g) [16,17]. Tagatose appears as a white crystal or powder, and it has a melting point of 134 °C [15]. It is a highly soluble ingredient and is stable at a pH range of 2–7 [16]. Tagatose has been reported to have similar physical and sensory characteristics as sugar, and it has been suggested to be used as a sugar replacer [18]. Fujimaru et al. [19] showed that tagatose elicits sweetness without any undesirable quality trait in aqueous solutions. Thus far, the evaluation of the technological and sensory effects of tagatose on yogurt products remains poorly studied. While tagatose has been declared as a GRAS (generally regarded as safe) ingredient for use in food products by the Food and Drug Administration (FDA) in the United States [20], more research studies on tagatose are needed given the lack of knowledge of its sensory and physico-chemical effects on food products. 

Therefore, the incorporation of tagatose as a sucrose replacer may offer desirable sensory characteristics in sugar-reduced products. The effect of sugar replacement with tagatose on the physico-chemical and sensory characteristics of low-sugar yogurt is yet to be studied. Indeed, a healthier yogurt alternative cannot outweigh its sensory qualities, and its acceptance is dependent on the degree of the consumers’ satisfaction [21,22]. An understanding of its functionality and its influence on consumers perception are critical for tagatose to successfully replace sugar in yogurt products. Thus, the objective of this study was to evaluate the effects of tagatose as a sugar substitute on selected physico-chemical properties and the sensory acceptability of strawberry-flavored yogurts.

## 2. Materials and Methods

### 2.1. Materials 

The ingredients used in the experimental yogurt preparation included full-cream fresh milk (Coles Supermarkets Australia Pty Ltd., Hawthorn East, VIC, Australia), full-cream milk powder (Devondale Murray Goulburn, Southbank, VIC, Australia), mild yogurt culture including *Lactobacillus delbrueckki* ssp. *Bulgaricus*, and *Streptococcus thermophilus* (Green Living Australia, Underwood, QLD, Australia), caster sugar (Coles Supermarkets Australia Pty Ltd., Hawthorn East, VIC, Australia), D-tagatose (NuNaturals, Eugene, OR, USA), and fresh strawberries (Coles Supermarkets Australia Pty Ltd., Hawthorn East, VIC, Australia). 

### 2.2. Preparation of Strawberry-Flavored Yogurt Samples

**Figure 1:**
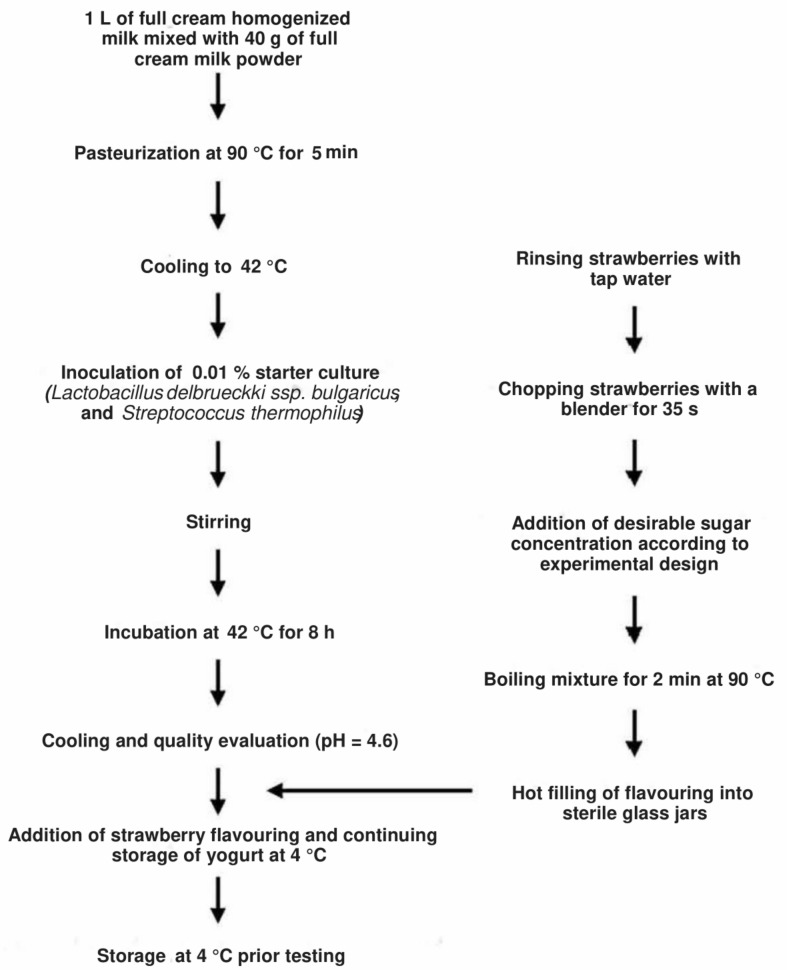
Flow chart of steps involved in the preparation of strawberry-flavored yogurt samples (Extracted from Torrico, et al. [23]).

Strawberry yogurt samples were produced at the Sensory Laboratory belonging to the University of Melbourne, School of Agriculture and Food, Parkville, Australia. For the preparation of the strawberry-flavoring jam, fresh strawberries were liquefied using a blender (BL480 Auto-IQ One Touch Intelligence, Nutri Ninja, Boston, MA, USA) for 35 s, and the desired sucrose concentration was added for each treatment (Table 1). A concentration of 5.5% strawberry jam was used due to its correspondence to the average strawberry-flavoring concentration found in Australian markets. Pasteurization of each strawberry-flavoring treatment was performed for 2 min at 90 °C using a cooking pot on a commercial stove. Strawberry-flavorings were hot-filled into sterile glass jars and were stored at 4 °C for 12 h (Figure 1). 

Mild yogurt was prepared by mixing 40 g of full-cream milk powder with 1 L of full-cream milk, and then pasteurized at 90–95 °C for 5 min (Figure 1). Pasteurized milk was cooled down to 42–45 °C, and the freeze-dried starter cultures containing *Lactobacillus delbrueckki* ssp. *Bulgaricus* and *Streptococcus thermophilus* were inoculated according to the manufacturer recommendations (0.001 g per 1 L of yogurt). The mix was transferred immediately to the yogurt maker (Greek yogurt and cheese maker, Kuvings Australia, Croydon, NSW, Australia) and incubated for 8 h at 42 °C. Quality evaluation of the yogurt was made to ensure the pH reached 4.6. Samples were then poured into 1.5 L plastic container with lids (Woolworths Group, Bella Vista, NSW, Australia) and transferred to a cold room ≤4 °C for 12 h. The strawberry jam and yogurt were blended on the next day and stored at 4 °C for consumer and physico-chemical tests. The strawberry-flavored yogurt was prepared in one lot for the physicochemical and consumer tests. Strawberry-flavored yogurts used for the physico-chemical analyses were stored in sterile plastic containers with lids at 4.5 ± 1.0 °C for 28 days for the shelf-life evaluation.

The range of sucrose (sugar)/tagatose concentrations evaluated in this experiment was determined in previous focus group discussions (*n* = 6) within the sensory group of The University of Melbourne, in which overall product attitudes/acceptability, and sweetness and sourness intensities were evaluated and discussed by panelists. Six sucrose/tagatose concentrations were prepared with equidistant decrements of sucrose (8.50, 6.80, 5.10, 3.40, 1.70, and 0.00 g/100 g of yogurt), and their respective equidistant increments of tagatose (0.0, 1.85, 3.70, 5.54, 7.39, and 9.24 g/100 g of yogurt) to determine the most accepted sucrose/tagatose concentration in the strawberry-flavored yogurts (Table 1). The 8.5%-sucrose concentration was selected as the starting point because it was the most preferred concentration of sucrose in strawberry-flavored yogurt from a preliminary study [23]. 

### 2.3. Physico-Chemical Analysis

Physico-chemical analyses (pH, lactic acid (%), °Brix, water holding capacity, viscosity, and color) were measured in the yogurt samples on the 1st, 7th, 14th, 21st, and 28th day of storage at 4 ± 1 °C. The pH of yogurt samples was measured at room temperature using a pH meter (Benchtop pH/mV meter, 860031, Sper scientific direct, Scottsdale, AZ, USA). The pH meter was calibrated with fresh pH standard buffers (4.0 and 7.0). Titratable acidity (TA; lactic acid) was measured by the methods of Shori et al. [24]. Approximately 10.0 g of yogurt sample were diluted with an equal amount of Milli-Q water (Millipore, Bedford, MA, USA) and titrated with 0.1 M NaOH using a 0.5% phenolphthalein indicator to reach the end point of a faint pink color. The TA was expressed as a percent of lactic acid based on the sample weight using the following formula: (Lactic acid (%) = *V* × 0.009/*W* × 100), where *V* is the volume of 0.1 M NaOH (mL) and *W* is the weight of yogurt (g). Titratable acidity was determined using the average of three replicates per sample. The total soluble solids content was measured with a digital refractometer for °Brix determination (HI96801, Hanna instruments, Melbourne, VIC, Australia). The refractometer was calibrated using distilled water. Mean values from six replicates and standard deviations were calculated. The water holding capacity (WHC) of yogurt was determined using a refrigerated centrifuge (Allegra X-12R, Beckman Coulter, Indianapolis, IN, USA). Yogurt samples (5 g) were centrifugated at 4500× *g* (relative centrifugal force, RCF) for 15 min at 4 °C. After centrifugation, the clear supernatant that formed was collected and weighed. Triplicates were measured for each sample and averaged. The extent of the whey separation of yogurt samples was calculated from the weight of the supernatant and yogurt following Equation (1):

(1) $$\mathrm{WHC}\left(\%\right)=\;\left(1-\frac{\mathrm{Supernatant}\;\mathrm{Weight}\;\left(g\right)}{\mathrm{Yogurt}\;\mathrm{Weight}\;\left(g\right)}\right)\times 100$$

Furthermore, the viscosity of the yogurt samples was measured using a Brookfield viscometer (model DV-II+, AMETEK Brookfield, Middleborough, MA, USA). For this, 50 g of yogurt sample was retrieved from the storage container and placed into a 50 mL beaker. All samples were placed on chilled ice to maintain the storage temperature (4 ± 1 °C). The viscometer was operated at 20 revolutions per minute (RPM) (spindle number 4) [9]. The viscosity values were expressed as centipoises (cP) and recorded after 40 s of rotation. All samples were allowed to rest for 60 s after each recording to eliminate the effect of immediate time dependence. All determinations were repeated six times on the same batch of the sample, and the average value and standard deviation of six measurements were recorded. A colorimeter (WR 10, FRU, Shenzhen, China) was used to determine lightness (L*), red/greenness (a*), and yellow/blueness (b*) values of the strawberry-flavored yogurt samples. A standard white tile was used to standardize the instrument. The color parameters (L*, a*, and b*) were measured three times and averaged on the surface of each yogurt treatment.

### 2.4. Sensory Evaluation

#### 2.4.1. Subjects

All sensory testing protocols were listed as minimal risks with the ethics approval 1,543,704.2 in February 2017 by the Human Ethics Advisory Group (HEAG) of the Faculty of Veterinary and Agricultural Science at The University of Melbourne, Australia. A total of *n* = 55 untrained participants (38 females and 17 males, aged 18 to 45 years old) were recruited from a pool of staff and students at The University of Melbourne, Australia, via the university noticeboard. According to the power analysis for this study, the number of consumers (*n* = 55) was sufficient to find significant differences (1 − β > 0.99) among the different yogurt treatments in the acceptability test. This was calculated using the Statistical Analysis Software SAS^®^ (version 9.4, SAS Institute Inc., Cary, NC, USA) for one-way ANOVA. Participants were pre-screened using the following criteria: (1) regular consumers of yogurt (at least once per month) based on self-reported responses, and (2) not having taste/smell disorders. Consumer evaluations took place in individual booths in the Sensory Laboratory at The University of Melbourne under a controlled environment with illuminated modern LED lights (configured with color white; RGB = 255, 255, 255) and a set room temperature of 25 °C. Before the tasting session, participants were required to sign a consent form approved by the HEAG (The University of Melbourne). All participants were also informed of any allergens that may be present in the yogurt samples. Therefore, all participants were healthy individuals who could consume yogurts regularly. Consumers who participated in the sensory evaluation were compensated with a chocolate bar at the end of the session.

#### 2.4.2. Sensory Procedure

Participants were asked to complete a consumer acceptance test on all six strawberry-flavored yogurt samples (Table 1). Each sample was poured into a 30 mL plastic cup coded with a three-digit random number. The presentation order of the samples was randomized within each participant. Yogurt samples were evaluated with an internal temperature of 4 ± 1 °C. Participants were asked to evaluate the liking of appearance, color, glossiness, aroma, sweetness, sourness, aftertaste, thickness, smoothness, and overall liking of each sample using a nine-point hedonic scale (1 = dislike extremely, 5 = neither like nor dislike, 9 = like extremely) and rate the relative intensity of sweetness, sourness, strawberry flavor, and thickness using a three-point just about right (JAR) scale (1 = too little, 2 = just about right, 3 = too much). The purchase intent (Question: Would you purchase this product if it was available at a reasonable price where you normally shop?) of each strawberry-flavored yogurt sample was determined using a binomial scale (1 = Yes, 2 = No). Two purchase intent questions were asked using the same questionnaire: the first question was assessed without any additional information (before), and the second question was asked after consumers had been informed that the product was reduced in sugar (Question: Would you purchase this product knowing that it has less sugar than regular strawberry-flavored yogurts?). At the end of the questionnaire, participants were asked to rank all samples according to their preference (1 = most preferred, 6 = least preferred). The design of the questionnaire and the gathering of responses were conducted using the Bio-Sensory application (The University of Melbourne). Sensory evaluations of the yogurt samples were made after one day of storage.

### 2.5. Experimental Design and Statistical Analysis

A completely randomized design (CRD) was used to investigate the effect of sugar reductions and tagatose replacements on the sensory properties of yogurt samples. A repeated-measurements design (RMD) was used to investigate the effects of sugar reduction on the physico-chemical parameters of yogurt samples during the 28 days of storage at 4 ± 1 °C. Analysis of variance (ANOVA) with a generalized linear model (GLM) and a post hoc Tukey’s honestly significantly different (HSD) test (*p* ≤ 0.05) were used to assess significant differences in the hedonic ratings and instrumental measurements of the strawberry-flavored yogurt samples. A penalty test on the JAR ratings was performed to determine the effects of the sensory attributes on the hedonic liking of yogurt samples. The total penalty score (TPS) for individual attributes was calculated by multiplying the percentage of “not-JAR” (either “too little” or “too much”) by the corresponding mean decrease (the difference between the liking score at “not-JAR” and the liking score at JAR). For the purchase intent data, multiple pairwise comparisons were performed using the Cochran’s *Q* test and the simultaneous confidence intervals testing. The McNemar test was used to determine statistical differences in purchase intent before and after the reduced sugar information was provided to consumers. The Friedman analysis was performed for the preference and ranking data. A principal component analysis (PCA) was applied to assess relationships between overall liking and the physico-chemical data of the yogurt samples at day 1. A product-attribute bip-lot was used for illustration of the PCA. Hierarchical cluster analysis (HCA) was performed using the Euclidean distance, and the Wards linkage was used to categorize sample groups that were similar in the sensory and analytical results. Data analyses were performed using the XLSTAT statistical software, version 2017(Addinsoft, New York, NY, USA).

## 3. Results

### 3.1. Physico-Chemical Properties of Tagatose-Substituted Strawberry-Flavored Yogurts

The pH, lactic acid (%), °Brix, water holding capacity (WHC), and viscosity measurements of the strawberry-flavored yogurt samples substituted with different percentages of tagatose are shown in Table 2. The samples with 20–40% tagatose substitution had significantly (*p* < 0.05) higher pH values compared to those of samples with 80–100% tagatose substitution at day 1 (4.30–4.31 vs. 4.29, respectively). 

The pH values of all samples, disregarding the level of tagatose substitution, decreased significantly (*p* < 0.05) after 28 days of storage at 4 ± 1 °C (from 4.29–4.31 at day 0 to 4.20–4.23 at day 28). At the end of the storage period (day 28), the 100% sucrose sample had the lowest pH value compared to those of the other treatments (4.20 vs. 4.21–4.23, respectively). The titratable acidity values for the tagatose-substituted and 100% sucrose samples, which are expressed as lactic acid (%), were not significantly different (*p* ≥ 0.05) regardless of the concentration of tagatose at day 1 (0.94–0.96). The lactic acid (%) of the 100% sucrose treatment was not different (*p* ≥ 0.05) than other yogurt treatments throughout the storage period (Table 2). A significantly (*p* < 0.05) higher lactic acid (%) value in the 100% sucrose sample was found when compared to the Suc (40%)/Tag (60%) and Suc (20%)/Tag (80%) samples on the 21st (1.14 vs. 1.10–1.09, respectively) and 28th days of storage (0.13 vs. 0.09–0.08, respectively). The lactic acid (%) values of all samples significantly (*p* < 0.05) increased during the first 14 days of the storage period; after that, those values stayed somewhat stabilized (*p* ≥ 0.05) for the remaining time. 

The °Brix values of the strawberry-flavored yogurts with any level of tagatose substitution were not significantly (*p* ≥ 0.05) different at day 1 (16.91–17.80; Table 2). The 28 days of storage period showed minimal effects (*p* ≥ 0.05) on the °Brix values of the samples (16.91–17.80 at day 1 to 17.33–18.01 at day 28). The ratio of sucrose/tagatose showed no noticeable effect (*p* ≥ 0.05) on the WHC values of the yogurt samples at day 1 (70.46–72.29%). Besides, all samples had similar WHC values after 28 days of storage (68.13–71.37%). However, the 100% sucrose samples had a significantly (*p* < 0.05) lower WHC value compared to that of the 20% tagatose sample after 28 days of storage (68.49% vs. 71.37%, respectively). Tagatose substitution showed minimal effects on the viscosity of yogurt samples. All samples except Suc (80%)/Tag (20%) obtained similar (*p* ≥ 0.05) viscosity values compared to that of the 100% sucrose sample (6728.33–8123.33 cP vs. 7523.33 cP, respectively). The viscosity values of all samples decreased significantly (*p* < 0.05) after 28 days of storage at 4 ± 1 °C (from 6728.33–8123.33 cP at day 1 to 4171.67–4863.33 cP at day 28).

Table 3 shows the lightness (L*), red/greenness (a*), and yellow/blueness (b*) values of the strawberry-flavored yogurt samples with various degrees of sucrose reduction and tagatose substitution. The replacement of sugar with tagatose showed marginal effects on the L* values of the yogurt samples. However, the 100% tagatose sample was generally higher in L* compared to the 100% sucrose sample throughout the 28 days of storage time, with significant (*p* < 0.05) differences found in the 1st and 7th days (Table 3). The L* value of the samples Suc (100%), Suc (80%)/Tag (20%), and Suc (60%)/Tag (40%) significantly increased (*p* < 0.05) after 28 days of storage. The a* values of the yogurt samples decreased significantly (*p* < 0.05) with the increasing substitution of sucrose by tagatose at day 1 (from 0.72 of the 100% sucrose sample to 0.37 of the Suc (60%)/Tag (40%) sample, and to −0.34 of the 100% tagatose sample). The treatments with higher sucrose concentrations had higher a* values during the entire storage time (Table 3). Upon 28 days of storage, the a* values of all samples decreased significantly (*p* < 0.05) from between −0.34 and 0.72 at day 1 to between −0.96 and −0.23 at day 28. Tagatose substitutions showed no clear pattern on the b* values of the formulated yogurt samples. However, the b* values of the 100% tagatose samples were significantly (*p* < 0.05) higher than the values of the 100% sucrose samples throughout the storage time (7.13–7.54 vs. 6.46–6.92). After 28 days of storage, the b* values of the 100% sucrose and the Suc (40%)/Tag (60%) samples increased significantly (*p* < 0.05) from 6.46–6.81 at day 1 to 6.92–7.37 at day 28.

### 3.2. Sensory Evaluation of Tagatose-Substituted Strawberry-Flavored Yogurts

#### 3.2.1. Acceptability of Sensory Attributes in Tagatose-Substituted Strawberry-Flavored Yogurts

The effects of tagatose substitution on the sensory liking/acceptability of strawberry-flavored yogurts are summarized in Table 4. All treatments were similar in acceptability, with no significant (*p* ≥ 0.05) differences found on the liking scores of any attributes. However, the treatment with 100% tagatose was generally lower but not significant (*p* ≥ 0.05) in the liking scores of all attributes, except for color and appearance. Moreover, the liking scores of the 100% sucrose treatment were not significantly (*p* ≥ 0.05) different for the visual attributes of appearance, color, and glossiness when compared to other yogurt treatments.

#### 3.2.2. JAR Responses and Total Penalty Scores of Tagatose-Substituted Strawberry-Flavored Yogurts

**Figure 2:**
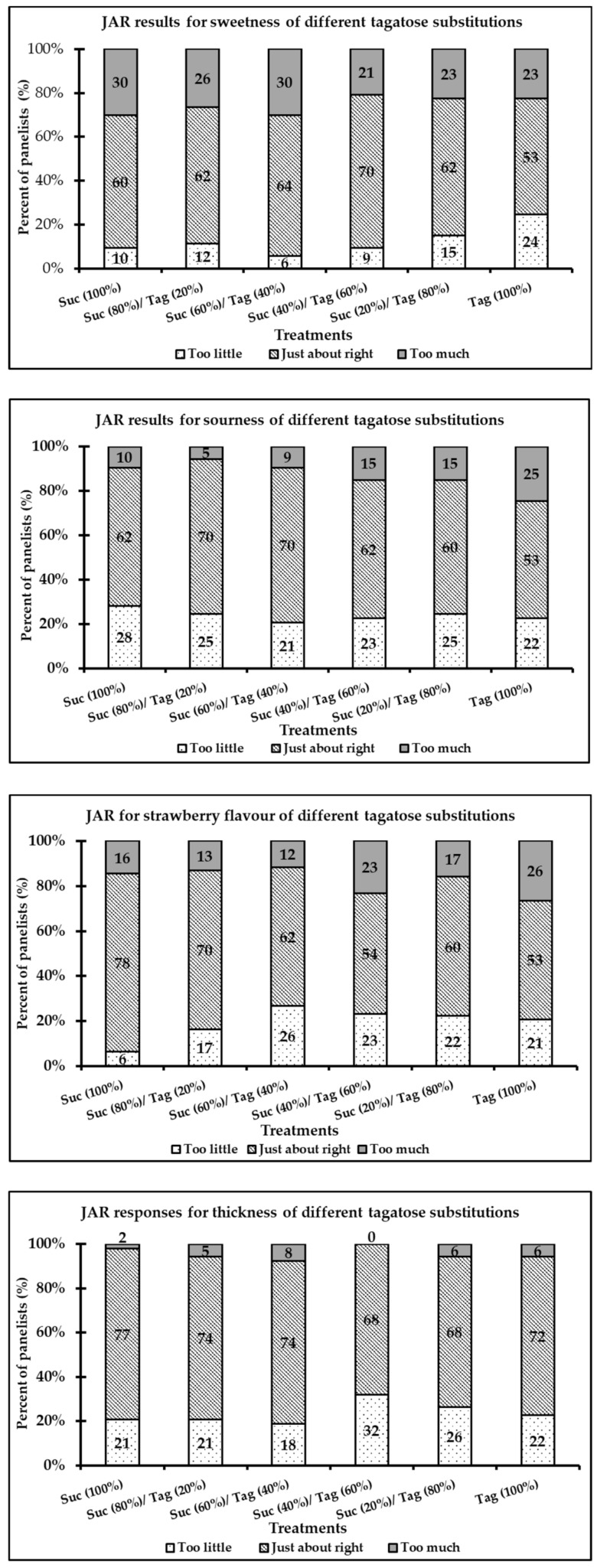
Selection frequencies (%) of the just-about-right (JAR) results in overall taste liking for sweetness, sourness, strawberry flavor, and thickness of strawberry-flavored yogurt samples (treatment labels are shown in Table 1).

The frequency ratings of sweetness, sourness, strawberry flavor, and thickness of the strawberry-flavored yogurt samples using a three-point JAR scale are illustrated in Figure 2. For all treatments, the selection of just-about-right for all attributes was found to be higher compared to the selections of “too much” or “too little.” In general, all samples were rated as just-about-right in sweetness (53–70%), sourness (53–70%), strawberry flavor (53–78%), and thickness (68–77%). Although slight changes in the JAR selections were observed for all yogurt samples, the 100% tagatose samples were marginally “too little” in sweetness (24% vs. 6–15%) and “too much” in sourness (25% vs. 5–15%) compared to the other treatments. 

**Figure 3:**
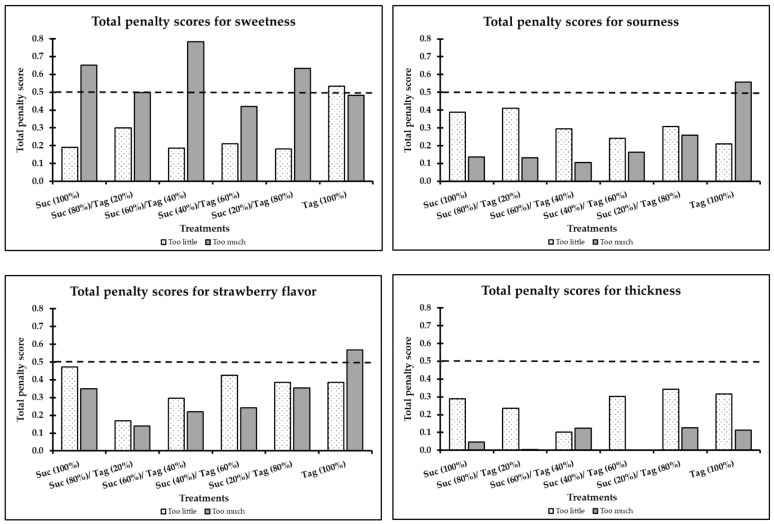
Total penalty scores in overall taste liking for sweetness, sourness, strawberry flavor, and thickness of strawberry-flavored yogurt samples (treatment labels are shown in Table 1).

On the other hand, the selection of “too little” in strawberry flavor was lower for the 100% sucrose sample compared to those values of the other treatments (6% vs. 17–26%). Figure 3 shows the total penalty scores in the overall liking of the strawberry-flavored yogurts according to the JAR deviations of sweetness, sourness, strawberry flavor, and thickness. Penalty analysis was only conducted on the attributes that obtained the skew cut-off percentage (>20%) for the “too much” or “too little” selection of the evaluated attributes [25]. Overall, the 100% tagatose samples were penalized as being considered “too much” in strawberry flavor and sourness (strawberry flavor TPS = 0.57 and sourness TPS = 0.56). For the thickness attribute, the total penalty scores for all yogurt treatments were lower than the critical value (0.50), which when combined with the JAR results, indicated that the participants did not consider the thickness to be “too little” nor “too much” (Figure 3). 

#### 3.2.3. Consumers Preference and Purchase Intent of Tagatose-Substituted Strawberry-Flavored Yogurts

Table 5 shows the purchase intent and ranking of the strawberry-flavored yogurts. Before the provision of the sugar reduced information (purchase intent before) to the participants, the purchase intent of yogurt with 100% sucrose was not significantly (*p* ≥ 0.05) different than those values of the tagatose-substituted treatments (64.14% vs. 43.40–60.38%). After the information of sugar reduction was provided (purchase intent after) to the participants, the samples Suc (20%)/Tag (80%) and 100% tagatose showed a significant (*p* < 0.05) increase in their purchase intent from their original values (from 43.40–52.83% before to 56.60–67.93% after; Table 5). The 100% tagatose sample, regardless of whether sugar reduction information was provided to the participants, was marginally (*p* ≥ 0.05) lower in purchase intent compared to the other yogurt treatments. 

In terms of preference (ranking data), the 100% tagatose sample received the highest rank sum value (228), which was significantly different compared to the 100% sucrose treatment (rank sum = 167). This indicates that the preference of the 100% tagatose sample was significantly (*p* < 0.05) lower compared to that of the 100% sucrose sample. However, the ranking value of the 100% sucrose sample was not significantly different compared to the yogurt treatments with partial substitutions of tagatose (167 vs. 177–186, respectively; Table 5).

### 3.3. Multivariate Analysis of Tagatose-Substituted Strawberry-Flavored Yogurts

**Figure 4:**
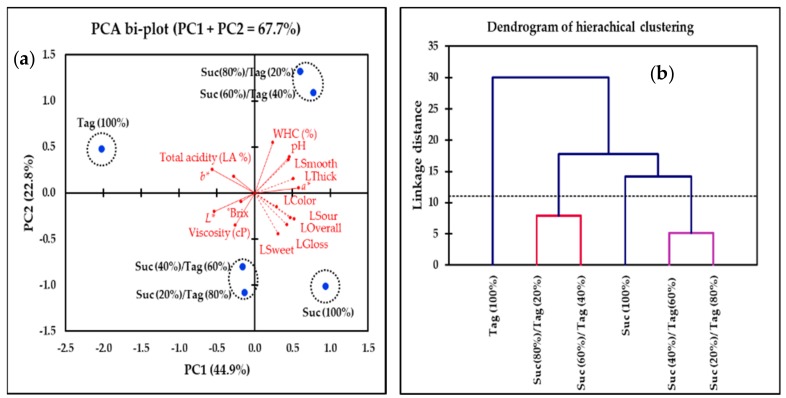
(**a**) Principal component analysis (PCA) bi-plot and (**b**) cluster analysis visualizing treatments (strawberry-flavored yogurt samples), liking attributes (dash line vectors; smoothness, thickness, color, sourness, glossiness, sweetness, and overall liking), and physico-chemical properties (solid line vectors; pH, total acidity, viscosity, water holding capacity, color, and °Brix) at day 1 of storage time (treatment labels are shown in Table 1).

The results obtained by the hierarchical cluster analysis of all strawberry yogurt samples considering the sensory (sweetness, sourness, glossiness, thickness, smoothness, and overall liking) and physico-chemical attributes (°Brix, pH, total acidity, water holding capacity, viscosity, and color) are presented in Figure 4. Four main cluster groups were identified: cluster 1 (Tag (100%)), cluster 2 (Suc (100%)), cluster 3 (Suc (40%)/Tag (60%) and Suc (20%)/Tag (80%)), and cluster 4 (Suc (80%)/Tag (20%) and Suc (60%)/Tag (40%)). As indicated by the linkage distance, the 100% tagatose sample had the largest cluster discrimination compared to that of the other treatments. The samples Suc (40%)/Tag (60%) and Suc (20%)/Tag (80%) had a shorter cluster distance to the 100% sucrose sample compared to that of the samples Suc (80%)/Tag (20%) and Suc (60%)/Tag (40%).

The PCA bi-plot (Figure 4) describes the interrelations of the physico-chemical and selected sensory variables with the strawberry-flavored yogurt treatments. The selection of the sensory variables showed in the bi-plot were based on their factor-loading contributions (factor loading values >0.5). The PCA bi-plot shows that 67.7% of the total variation was explained by the first two principal components (PC1 = 44.9% and PC2 = 22.8%). In terms of the sensory attributes, the arrangement of vectors in the bi-plot space shows a positive association in the liking of color, sourness, glossiness, sweetness, and overall liking. For the physico-chemical attributes, the viscosity, L* color, and °Brix parameters were positively associated with each other but negatively associated with WHC (%) and pH. Total acidity (lactic acid (%)) was positively linked with b* color and was marginally associated with the other physico-chemical parameters. Liking of smoothness and thickness were positively associated with WHC (%) and pH but were negatively associated with the L* color, viscosity, and °Brix. Total acidity and the b* color were negatively associated with the liking of color, sweetness, sourness, glossiness, and overall liking. In terms of the treatments, four strawberry yogurt groups were separated along PC1 and PC2, considering both sensory and physico-chemical attributes. The PCA result was consistent with the findings from the cluster analysis. The 100% tagatose sample was strongly associated with the total acidity and b* color value, while the 100% sucrose sample was linked mostly with the liking of glossiness, sourness, sweetness, and overall liking.

## 4. Discussion

### 4.1. Physico-Chemical Properties of Tagatose-Substituted Strawberry-Flavored Yogurts

Sucrose replacement with tagatose showed marginal (*p* ≥ 0.05) effects on the pH values of the yogurt samples (Table 2). This can be attributed to the similar consumption of D-tagatose and sucrose by the lactic acid bacteria (*L. bulgaricus* and *S. thermophilus*) [26]. The significant decrease of pH (*p* < 0.05) after 28 days of storage at 4 °C may be associated with the continuing production of lactic acid from sugars [26]. Our results are similar to findings from previous studies that showed a significant decrease in pH of yogurt samples after extended storage periods [9,27]. The tagatose-substituted samples during the 28 days of storage were within the acceptable range of pH (4.0–4.5) according to Kroger [28]; thus, the microbiological quality of strawberry yogurt samples was maintained.

Titratable acidity measures the total acid concentration in foods using the titration method with a strong base to an endpoint [29]. According to Belitz and Grosch [30], the effect of titratable acidity on the sourness perception is more prominent compared to the effect of pH in foods. In a sensory study conducted on chocolate milk with added tagatose, an increase of the titratable acidity from 27.6 to 33.2 °D was associated with unpleasant sourness and sensory characteristics [20]. According to the results of the present study, tagatose substitution showed no significant effects (*p* ≥ 0.05) on the titratable acidity (measured as a percentage of lactic acid) of the yogurt samples. Moreover, the liking of sourness of the 100% sucrose sample was not significantly (*p* < 0.05) different compared to those of the samples that had tagatose in their formulations (Table 4). These results showed that the sensory quality associated with the titratable acidity was not affected by the tagatose replacements in yogurts. The lactic acid (%) of the samples increased significantly (*p* < 0.05) during the storage time, which had the potential effect of compromising the sensory characteristics of the yogurt. The present study was limited to associating the sensory and physio-chemical results for day 1 of storage. Further work has to be done to elucidate the effect of titratable acidity on the sensory quality of tagatose-substituted yogurts during extended storage periods.

The total soluble content (°Brix values) in tagatose-substituted samples was not affected by the replacement of tagatose (Table 2). This may be attributed to the similar physical properties of tagatose compared to sucrose [18]. The °Brix values varied during the storage time without an identifiable pattern for all samples. These discrepancies may be attributed to the susceptibility of tagatose to degradation. According to Kwon and Baek [31], tagatose as a reducing sugar is susceptible to degradation under various conditions. In the present study, the degradation of tagatose may have occurred during the heat treatment of the strawberry jam in the process of making the yogurt.

Water holding capacity is an important attribute that measures the ability of the food structure to retain water in its protein matrix [32]. In general, the WHC of all yogurt samples were not significantly (*p* ≥ 0.05) different (Table 2). These results suggested that the stability of coagulation in the tagatose-substituted samples was maintained. Similar to the results of the °Brix values, sugar-blended samples showed no obvious pattern in WHC (%). Marginal differences in the WHC (*p* ≥ 0.05) over the 28 days of storage period suggested that there was a minimal degradation of the protein matrix in the tagatose-substituted yogurt samples.

In the present study, the viscosity of Tag (100%) was not significantly (*p* ≥ 0.05) different compared to that of Suc (100%) (Table 2). Similar to the findings in the present study, Shourideh et al. [33] found that a 100% tagatose dark chocolate sample had a similar apparent viscosity compared to the value of a 100% sucrose sample. Parallel to our results, the viscosity values decreased significantly for all the yogurt samples after 28 days of storage at 4 °C [23]. Stabilizers, such as polysaccharides or gelatin, can avoid the whey separation in yogurt products [34]. An appropriate amount and type of stabilizer may be incorporated in future formulations of tagatose-substituted yogurts for the improvement of the consistency and stability throughout the storage period.

There were differences in color (CieLab color code) among the tagatose-substituted strawberry yogurts during the 28 days of storage period (Table 3). The tagatose-substituted samples were significantly lower in the a* value (less red) compared to that of the 100% sucrose sample. Moreover, the 100% tagatose had significantly (*p* < 0.05) higher L* and b* values, and a lower a* value compared to those of the 100% sucrose strawberry-flavored yogurt. This may be attributed to the relatively lower solubility of tagatose compared to sucrose [18]. In the present study, tagatose might have retained its slight indissoluble nature in the mixing of the strawberry yogurts. This can produce a particle size increase of the soluble ingredients in the yogurt samples that may have also altered the light-refracting properties [35]; thus, affecting the color. During the 28 days of storage, the b* and L* values increased, and the a* value decreased for all yogurt samples. This effect can potentially be attributed to the compositional changes in the yogurt samples during the storage time [26].

### 4.2. Sensory Evaluation of Tagatose-Substituted Strawberry-Flavored Yogurts

The acceptability of nine sensory attributes (appearance, color, glossiness, aroma, sweetness, sourness, aftertaste, thickness, and smoothness), and JAR questions of four attributes (sweetness, sourness, strawberry flavor, and thickness) were evaluated for the 100% sucrose and all tagatose-substituted strawberry yogurt samples. Tagatose substitutions showed no significant effects on the acceptability of all attributes compared to that of the 100% sucrose yogurt. Besides, the just-about-right selection accounted for the largest percentage of consumer perception of sweetness, sourness, strawberry flavor, and thickness for all samples (Figure 2). These results are somewhat expected due to the similar sensory and physical characteristics of tagatose compared to sucrose [16,17]. Tagatose appeared to mostly affect the relative intensity of the strawberry flavor, in which a greater variation of the JAR frequencies (53–78%) was found across the tagatose-substituted yogurt treatments. The JAR selection for the strawberry flavor was higher for the 100% sucrose sample (78%) compared to that of the 100% tagatose sample (53%). Sugar and fruity or floral aromas interact with each other in the form of potentiation [36]. For the yogurt samples in the present study, the sugar may have potentially enhanced the strawberry flavor at the time of tasting. The results of the present study showed that there was a differential effect of tagatose and sucrose on the flavor perception despite the equal level of sweetness that was used for preparing the samples. Further investigations on the effect of tagatose on flavor release are needed to study these effects. 

In terms of acceptability, the different formulation ratios of sucrose/tagatose had no significant effect (*p* ≥ 0.05) on the liking of any attribute. However, the 100% tagatose sample had consistently lower liking scores (but not significant) for all attributes compared to those of the other treatments (Table 4). The lower acceptability levels of the 100% tagatose sample in sweetness, sourness, and strawberry flavor may be potentially related to a different perceived intensity level of these attributes. For instance, the 100% tagatose sample was significantly penalized (TPS > 0.5) for being “too little” in sweetness (TPS = 0.53), “too much” in sourness (TPS = 0.56), and “too much” in strawberry flavor (TPS = 0.57) (Figure 3). 

Previous studies have shown inconclusive effects of providing health information on consumer choice for various products [37,38]. The information related to sugar reduction (that was provided to the participants) positively affected the purchase intent of the yogurt products. All samples sweetened with tagatose increased their purchase intent after receiving this information, particularly, for the samples that had lower attribute liking scores (Tag (100%) and Suc (20%)/Tag (80%)) (Table 5). In terms of the energy value, sugar (sucrose) has 1700 kJ per 100 g of product. Tagatose, on the other hand, has an energy value of 630 kJ per 100 g of product [16,17]. These results potentially showed the importance of low-sugar labeling on consumers’ food choices. 

### 4.3. Multivariate Analysis of Tagatose-Substituted Strawberry-Flavored Yogurts

Yogurt with 100% sucrose was the most preferred sample treatment for the participants; however, the acceptability of all the samples was not significantly different (Table 4 and Table 5). In contrast, the hierarchical cluster analysis distinguished a unique sensory and physico-chemical profile of the 100% tagatose sample compared to those of the other yogurt treatments. Interestingly, the overall quality (in terms of attribute liking and physico-chemical measurements) of the Suc (40%)/Tag (60%) and Suc (20%)/Tag (80%) was more similar to the 100% sucrose sample compared to that of the Suc (80%)/Tag (20%) and Suc (60%)/Tag (40%). These results suggested that there were different interaction effects between the tagatose and sucrose among the tagatose-substituted samples. Moreover, it was possible that there were other critical factors (ingredients and processing) other than the sugar ratio that can potentially contribute to the variations of the physico-chemical interactions in the yogurt. Presumably, the heating treatment used for the strawberry jam may have also affected the tagatose and sucrose differently due to their differences in melting point (tagatose: 134–135 °C vs. sucrose: 160–186 °C) [3]. Future studies should focus on adjusting the tagatose heating temperatures and times in the preparation of jams for yogurts. 

A PCA bi-plot (Figure 4) showed the association of samples with various sensory and physico-chemical properties. The acceptability of the 100% sucrose sample was associated with the higher liking scores of sweetness, sourness, and glossiness. On the other hand, the tagatose-substituted samples were associated with the liking of smoothness and thickness. Interestingly, the liking scores of smoothness and thickness were positively associated with the samples Suc (80%)/Tag (20%) and Suc (60%)/Tag (40%), but negatively associated with the samples Suc (20%)/Tag (80%) and Suc (40%)/Tag (60%). Such negative association was related to the higher °Brix and viscosity values of the samples Suc (20%)/Tag (80%) and Suc (40%)/Tag (60%). 

Participants in the present study may have disliked the slightly thicker and smoother texture of the samples Suc (20%)/Tag (80%) and Suc (40%)/Tag (60%). The 100% tagatose sample was negatively associated with the liking of sourness (liking score = 5.67), glossiness (liking score = 6.23), and color (liking score = 6.19) (Table 4, Figure 4). The relatively lower acceptability of glossiness and color in the 100% tagatose sample may be attributed to a decrease in the red color (a* value; −0.34 vs. 0.72 of the 100% sucrose sample) and an increase in yellowness (b* value; 7.13 vs. 6.46 of the 100% sucrose sample), while the lower liking of sourness may be related to the slightly lower pH value reported for the 100% tagatose yogurt (4.29 vs. 4.30 of the 100% sucrose sample; Table 2 and Table 3). Further studies should be dedicated to evaluating the effects of changes in pH on the physico-chemical and sensory properties of yogurt with tagatose replacements.

## 5. Conclusions

Strawberry yogurts with tagatose replacements had similar acceptability scores for appearance, color, glossiness, aroma, sweetness, sourness, aftertaste, thickness, and smoothness. Sucrose reductions by the replacement of up to 80% tagatose showed marginal effects on the acceptability and preference of the strawberry yogurts; although the loss of red color (a* value) and the increase in yellowness (b* value) in the tagatose-substituted yogurts was significant. Sucrose reduction showed a positive effect on the purchase intent of the strawberry yogurts. Future studies should focus on the detailed understanding of the tagatose–sucrose interactions for the formulations of reduced-sugar yogurts. Sensory evaluations on tagatose-substituted strawberry-flavored yogurts during extended storage periods should be conducted for better inferences regarding the overall changes in perception. Biochemical and microbiological studies are also needed to examine the synergistic effects of tagatose and probiotics in yogurt products.

## Figures and Tables

**Table 1:** Formulations ^1^ of the different sucrose concentrations for the strawberry-flavored yogurt samples.

Treatment Label	Concentration of Sucrose	Concentration of Tagatose	Proportions of Sucrose and Tagatose ^1^
Suc (100%)	8.5 g/100 g yogurt	0.00 g/100 g yogurt	100% Sucrose, 0% Tagatose
Suc (80%)/Tag (20%)	6.8 g/100 g yogurt	1.85 g/100 g yogurt	80% Sucrose, 20% Tagatose
Suc (60%)/Tag (40%)	5.1 g/100 g yogurt	3.70 g/100 g yogurt	60% Sucrose, 40% Tagatose
Suc (40%)/Tag (60%)	3.4 g/100 g yogurt	5.54 g/100 g yogurt	40% Sucrose, 60% Tagatose
Suc (20%)/Tag (80%)	1.7 g/100 g yogurt	7.39 g/100 g yogurt	20% Sucrose, 80% Tagatose
Tag (100%)	0.0 g	9.24 g/100 g yogurt	100% Tagatose

^1^ The yogurt sample with a sucrose concentration of 8.5 g/100 g represented the full sucrose sample (100%). The subsequent sucrose concentrations represent reductions from the initial sucrose concentration. The sucrose/tagatose combinations were mixed with the pasteurized strawberry flavoring jam (5.5%) and blended into the pre-made yogurt. Abbreviations: Suc = sucrose, Tag = tagatose.

**Table 2:** Effects of tagatose substitutions on pH, lactic acid (%), °Brix, water holding capacity (WHC), and viscosity of yogurt samples during 28 days of storage at 4 °C.

Parameters	Treatments	Time of Storage (Days)				
		1	7	14	21	28
pH	Suc (100%)	4.30 ± 0.01 ^ab^	4.27 ± 0.01 ^c^	4.24 ± 0.00 ^efgh^	4.22 ± 0.01 ^ijk^	4.20 ± 0.00 ^l^
	Suc (80%)/Tag (20%)	4.31 ± 0.01 ^a^	4.26 ± 0.01 ^cd^	4.23 ± 0.01 ^hijk^	4.24 ± 0.01 ^fghi^	4.23 ± 0.01 ^hijk^
	Suc (60%)/Tag (40%)	4.31 ± 0.01 ^a^	4.26 ± 0.00 ^cd^	4.25 ± 0.00 ^def^	4.23 ± 0.00 ^hij^	4.22 ± 0.01 ^ijk^
	Suc (40%)/Tag (60%)	4.30 ± 0.01 ^ab^	4.25 ± 0.00 ^cde^	4.25 ± 0.01 ^defg^	4.23 ± 0.01 ^ghi^	4.23 ± 0.01 ^hijk^
	Suc (20%)/Tag (80%)	4.29 ± 0.01 ^b^	4.25 ± 0.01 ^cde^	4.25 ± 0.01 ^defg^	4.23 ± 0.00 ^hij^	4.23 ± 0.01 ^hijk^
	Tag (100%)	4.29 ± 0.01 ^b^	4.25 ± 0.01 ^cde^	4.25 ± 0.01 ^defg^	4.22 ± 0.01 ^jk^	4.21 ± 0.01 ^kl^
Lactic Acid (%)	Suc (100%)	0.96 ± 0.00 ^l^	1.06 ± 0.01 ^fghijk^	1.11 ± 0.02 ^abcde^	1.14 ± 0.01 ^a^	1.13 ± 0.01 ^ab^
	Suc (80%)/Tag (20%)	0.95 ± 0.02 ^l^	1.05 ± 0.01 ^ijk^	1.11 ± 0.01 ^abcde^	1.12 ± 0.01 ^abcd^	1.13 ± 0.00 ^ab^
	Suc (60%)/Tag (40%)	0.95 ± 0.01 ^l^	1.06 ± 0.01 ^fghijk^	1.11 ± 0.01 ^abcde^	1.13 ± 0.01 ^ab^	1.11 ± 0.00 ^abcde^
	Suc (40%)/Tag (60%)	0.94 ± 0.02 ^l^	1.04 ± 0.01 ^jk^	1.10 ± 0.00 ^bcdefg^	1.10 ± 0.01 ^bcdefg^	1.09 ± 0.00 ^cdefgh^
	Suc (20%)/Tag (80%)	0.94 ± 0.01 ^l^	1.06 ± 0.02 ^ghijk^	1.08 ± 0.01 ^efghi^	1.09 ± 0.01 ^defghi^	1.08 ± 0.00 ^efghij^
	Tag (100%)	0.96 ± 0.97 ^l^	1.06 ± 0.01 ^hijk^	1.08 ± 0.02 ^efghi^	1.10 ± 0.01 ^abcdef^	1.12 ± 0.01 ^abc^
°Brix	Suc (100%)	17.28 ± 0.24 ^gh^	17.91 ± 0.28 ^abcde^	17.28 ± 0.17 ^gh^	17.68 ± 0.33 ^bcdfeg^	17.68 ± 0.50 ^bcdfeg^
	Suc (80%)/Tag (20%)	17.80 ± 0.34 ^abcdefg^	17.76 ± 0.40 ^abcdfeg^	17.79 ± 0.24 ^abcdefg^	17.86 ± 0.48 ^abcdef^	17.33 ± 0.26 ^fgh^
	Suc (60%)/Tag (40%)	16.91 ± 0.20 ^h^	17.83 ± 0.38 ^abcdef^	17.62 ± 0.12 ^cdefg^	18.23 ± 0.37 ^a^	17.67 ± 0.17 ^bcdfeg^
	Suc (40%)/Tag (60%)	17.44 ± 0.31 ^efgh^	17.66 ± 0.36 ^cdefg^	18.07 ± 0.23 ^abc^	17.67 ± 0.34 ^bcdfeg^	17.66 ± 0.21 ^cdefg^
	Suc (20%)/Tag (80%)	17.78 ± 0.44 ^abcdefg^	18.20 ± 0.17 ^ab^	17.98 ± 0.26 ^abcde^	18.04 ± 0.25 ^abc^	18.01 ± 0.26 ^abcd^
	Tag (100%)	17.50 ± 0.27 ^defg^	17.84 ± 0.25 ^abcdef^	18.13 ± 0.20 ^abc^	17.73 ± 0.24 ^abcdfeg^	17.82 ± 0.37 ^abcdef^
WHC (%)	Suc (100%)	71.12 ± 0.55 ^abcd^	71.69 ± 0.59 ^ab^	71.16 ± 0.66 ^abcd^	71.87 ± 0.50 ^ab^	68.49 ± 0.54 ^de^
	Suc (80%)/Tag (20%)	72.29 ± 0.48 ^ab^	70.61 ± 0.72 ^abcde^	72.82 ± 1.14 ^a^	71.40 ± 0.77 ^ab^	71.37 ± 0.59 ^ab^
	Suc (60%)/Tag (40%)	71.71 ± 0.44 ^ab^	71.26 ± 0.74 ^abc^	71.39 ± 1.84 ^ab^	71.81 ± 0.69 ^ab^	70.69 ± 0.62 ^abcde^
	Suc (40%)/Tag (60%)	70.46 ± 0.69 ^abcde^	70.61 ± 1.25 ^abcde^	71.39 ± 0.68 ^ab^	71.27 ± 1.50 ^abc^	70.95 ± 0.74 ^abcd^
	Suc (20%)/Tag (80%)	70.65 ± 0.26 ^abcde^	70.03 ± 0.21 ^bcde^	70.48 ± 0.86 ^abcde^	70.28 ± 0.54 ^abcde^	68.13 ± 0.99 ^e^
	Tag (100%)	71.09 ± 0.45 ^abcd^	71.16 ± 0.80 ^abcd^	70.81 ± 1.74 ^abcde^	72.59 ± 0.50 ^ab^	68.62 ± 0.54 ^cde^
Viscosity (cP)	Suc (100%)	7523.33 ± 788.53 ^abc^	6343.33 ± 765.60 ^bcde^	6620.00 ± 648.88 ^abcd^	6201.67 ± 655.42 ^bcdef^	4175.00 ± 409.08 ^h^
	Suc (80%)/Tag (20%)	5921.67 ± 601.18 ^def^	6358.33 ± 691.94^bcde^	6648.33 ± 847.10 ^abcd^	5736.67 ± 513.84 ^defg^	4388.33 ± 224.63.28 ^gh^
	Suc (60%)/Tag (40%)	6853.33 ± 624.62 ^abcd^	6535.000 ± 902.59 ^bcd^	6418.33 ± 609.51 ^bcd^	6001.67 ± 622.43 ^def^	4781.67 ± 451.28 ^fgh^
	Suc (40%)/Tag (60%)	8123.33 ± 903.56 ^a^	6750.00 ± 814.42 ^abcd^	6370.00 ± 872.56 ^bcde^	5756.67 ± 740.07 ^defg^	4863.33 ± 482.19 ^efgh^
	Suc (20%)/Tag (80%)	6728.33 ± 611.83 ^abcd^	5950.00 ± 540.59 ^def^	5675.00 ± 657.35 ^defgh^	5843.33 ± 580.33 ^defg^	4171.67 ± 504.28 ^h^
	Tag (100%)	7655.00 ± 899.73 ^ab^	6425.00 ± 827.71^bcd^	6125.00 ± 746.69 ^cdef^	5953.33 ± 812.69 ^def^	4206.67 ± 556.58 ^h^

^a–l^ Mean ± standard deviation values that share the same letter within the same parameter were not significantly different (*p* ≥ 0.05). Treatment labels are indicated in Table 1.

**Table 3:** Effects of tagatose substitutions on the color (L*, a*, and b*) values ^1^ of yogurts during 28 days of storage at 4 °C.

Parameters	Treatments ^2^	Time of Storage (Days)				
		1	7	14	21	28
L* value	Suc (100%)	85.59 ± 0.18 ^efg^	85.11 ± 0.70 ^gh^	85.87 ± 0.08 ^cdef^	85.75 ± 0.13 ^cdef^	85.97 ± 0.20 ^abcd^
	Suc (80%)/Tag (20%)	85.49 ± 0.68 ^fg^	84.21 ± 0.49 ^i^	85.84 ± 0.37 ^cdef^	86.03 ± 0.20 ^bcde^	86.07 ± 0.07 ^abc^
	Suc (60%)/Tag (40%)	84.96 ± 0.29 ^h^	85.96 ± 0.11 ^abcd^	85.49 ± 0.44 ^fg^	85.97 ± 0.21 ^abc^	85.74 ± 0.12 ^cdef^
	Suc (40%)/Tag (60%)	86.04 ± 0.37 ^bcde^	86.00 ± 0.23 ^abc^	86.05 ± 0.14 ^abcd^	86.55 ± 0.19 ^a^	85.92 ± 0.16 ^abcd^
	Suc (20%)/Tag (80%)	85.68 ± 0.33 ^def^	86.03 ± 0.16 ^bcde^	86.17 ± 0.18 ^abcd^	86.41 ± 0.09 ^ab^	85.72 ± 0.08 ^cdef^
	Tag (100%)	86.42 ± 0.23 ^ab^	86.19 ± 0.18 ^abc^	86.15 ± 0.09 ^abcd^	86.00 ± 0.18 ^bcde^	86.01 ± 0.11 ^bcde^
a* value	Suc (100%)	0.72 ± 0.02 ^a^	0.39 ± 0.06 ^c^	0.27 ± 0.07 ^cd^	0.09 ± 0.08 ^efg^	−0.23 ± 0.09 ^ijk^
	Suc (80%)/Tag (20%)	0.57 ± 0.05 ^b^	0.37 ± 0.08 ^c^	0.21 ± 0.01 ^de^	−0.26 ± 0.27 ^ijk^	−0.31 ± 0.05 ^ijk^
	Suc (60%)/Tag (40%)	0.37 ± 0.08 ^c^	0.05 ± 0.03 ^fg^	0.04 ± 0.07 ^fg^	−0.26 ± 0.07 ^ijk^	−0.49 ± 0.03 ^mno^
	Suc (40%)/Tag (60%)	0.15 ± 0.04 ^def^	−0.14 ± 0.05 ^hi^	−0.18 ± 0.05 ^ij^	−0.40 ± 0.05 ^lmn^	−0.43 ± 0.07 ^lmn^
	Suc (20%)/Tag (80%)	−0.03 ± 0.06 ^gh^	−0.35 ± 0.02 ^klm^	−0.36 ± 0.11 ^klmn^	−0.50 ± 0.03 ^no^	−0.69 ± 0.09 ^p^
	Tag (100%)	−0.34 ± 0.09 ^kl^	−0.58 ± 0.05 ^op^	−0.60 ± 0.06 ^op^	−0.62 ± 0.06 ^op^	−0.96 ± 0.06 ^q^
b* value	Suc (100%)	6.46 ± 0.11 ^n^	6.49 ± 0.31 ^n^	6.82 ± 0.08 ^hijklm^	6.88 ± 0.07 ^fghijkl^	6.92 ± 0.16 ^fghijkl^
	Suc (80%)/Tag (20%)	6.80 ± 0.19 ^ijklm^	7.03 ± 0.23 ^defghi^	6.84 ± 0.04 ^ghijklm^	6.99 ± 0.13 ^efghijk^	7.00 ± 0.07 ^defghij^
	Suc (60%)/Tag (40%)	6.72 ± 0.19 ^jklmn^	6.81 ± 0.34 ^ijklm^	7.14 ± 0.12 ^bcdef^	7.26 ± 0.10 ^bcde^	6.59 ± 0.11 ^mn^
	Suc (40%)/Tag (60%)	6.81 ± 0.03 ^ijklm^	6.90 ± 0.17 ^fghijkl^	7.09 ± 0.06 ^cdefgh^	7.27 ± 0.05 ^abcde^	7.37 ± 0.12 ^abc^
	Suc (20%)/Tag (80%)	6.74 ± 0.15 ^jklmn^	7.05 ± 0.08 ^defghi^	7.11 ± 0.25 ^cdefg^	7.38 ± 0.13 ^ab^	6.70 ± 0.17 ^lmn^
	Tag (100%)	7.13 ± 0.15 ^abcdefg^	7.13 ± 0.07 ^bcdef^	7.27 ± 0.08 ^abcd^	7.40 ± 0.14 ^ab^	7.54 ± 0.18 ^a^

^1^ Values represent means and standard deviations (SD) of at least three replicates. ^2^ Treatment labels are indicated in Table 1. ^a–q^ Mean values that share the same letter within the same parameter were not significantly different (*p* ≥ 0.05).

**Table 4:** Mean values ^1^ for the sensory acceptability scores of the strawberry-flavored yogurts.

**Treatments ^2^**	**Appearance**	**Color**	**Glossiness**	**Aroma**	**Sweetness**
Suc (100%)	6.43 ± 1.45 ^a^	6.57 ± 1.43 ^a^	6.51 ± 1.27 ^a^	6.38 ± 1.29 ^a^	6.19 ± 1.69 ^a^
Suc (80%)/Tag (20%)	6.04 ± 1.64 ^a^	6.21 ± 1.23 ^a^	6.28 ± 1.25 ^a^	6.26 ± 1.48 ^a^	6.02 ± 1.59 ^a^
Suc (60%)/Tag (40%)	6.25 ± 1.30 ^a^	6.26 ± 1.26 ^a^	6.36 ± 1.16 ^a^	6.11 ± 1.41 ^a^	6.00 ± 1.83 ^a^
Suc (40%)/Tag (60%)	6.25 ± 0.13 ^a^	6.04 ± 1.19 ^a^	6.30 ± 1.13 ^a^	6.28 ± 1.41 ^a^	6.40 ± 1.56 ^a^
Suc (20%)/Tag (80%)	6.30 ± 1.34 ^a^	6.25 ± 1.36 ^a^	6.38 ± 1.21 ^a^	6.49 ± 1.53 ^a^	6.40 ± 1.60 ^a^
Tag (100%)	6.13 ± 1.40 ^a^	6.19 ± 1.37 ^a^	6.23 ± 1.27 ^a^	6.08 ± 1.34 ^a^	5.67 ± 1.83 ^a^
**Treatments ^2^**	**Sourness**	**After-Taste**	**Thickness**	**Smoothness**	**Overall Liking**
Suc (100%)	6.30 ± 1.44 ^a^	6.40 ± 1.76 ^a^	6.15 ± 1.50 ^a^	6.38 ± 1.42 ^a^	6.68 ± 1.61 ^a^
Suc (80%)/Tag (20%)	6.13 ± 1.43 ^a^	6.45 ± 1.51 ^a^	6.17 ± 1.31 ^a^	6.66 ± 1.19 ^a^	6.49 ± 1.48 ^a^
Suc (60%)/Tag (40%)	6.11 ± 1.49 ^a^	6.26 ± 1.69 ^a^	6.42 ± 1.41 ^a^	6.55 ± 1.51 ^a^	6.15 ± 1.68 ^a^
Suc (40%)/Tag (60%)	6.30 ± 1.58 ^a^	6.25 ± 1.49 ^a^	6.17 ± 1.33 ^a^	6.25 ± 1.12 ^a^	6.30 ± 1.42 ^a^
Suc (20%)/Tag (80%)	6.15 ± 1.51 ^a^	6.25 ± 1.52 ^a^	6.11 ± 1.59 ^a^	6.28 ± 1.61 ^a^	6.43 ± 1.58 ^a^
Tag (100%)	5.66 ± 1.56 ^a^	5.68 ± 1.72 ^a^	5.89 ± 1.48 ^a^	6.21 ± 1.45 ^a^	5.94 ± 1.89 ^a^

^1^ Values are represented as mean and standard deviation (*n* = 55). The liking scores were based on a nine-point hedonic scale (1 = dislike extremely, 9 = like extremely). ^2^ Treatment labels are indicated in Table 1. ^a^ Mean values that share the same letter within the same variable were not significantly different (*p* < 0.05).

**Table 5:** Purchase intent and ranking sums of the strawberry-flavored yogurt samples.

Treatments ^1^	Purchase Intent (PI, %)		Ranking (Rank Sums)
	PI-Before	PI-After	Preference
Suc (100%)	64.14% ^a,A^	-	167 ^a^
Suc (80%)/Tag (20%)	58.49% ^a,A^	69.81% ^a,A^	179 ^ab^
Suc (60%)/Tag (40%)	60.38% ^a,A^	62.26% ^a,A^	177 ^ab^
Suc (40%)/Tag (60%)	58.49% ^a,A^	71.70% ^a,A^	186 ^ab^
Suc (20%)/Tag (80%)	52.83% ^a,A^	67.93% ^a,B^	178 ^ab^
Tag (100%)	43.40% ^a,A^	56.60% ^a,B^	228 ^b^

^a^ For the purchase intent results, percentage values that share the same superscripts within the same column were not significantly different (*p* ≥ 0.05; Cochran *Q* test and simultaneous confidence interval test). ^A–B^ For the purchase intent results, percentage values with the same letter within the same row were not significantly different (*p* ≥ 0.05; McNemar test). For the ranking results, rank sum values with the same superscripts ^(a-b)^ within the same column were not significantly different (*p* ≥ 0.05; Friedman test). ^1^ Treatment labels are indicated in Table 1.
